# Myopericarditis: A Diagnosis of Uncertainty

**DOI:** 10.14740/cr428w

**Published:** 2015-10-25

**Authors:** Rafay Khan, Nneka Iroka, Sunil Tulpule, Sabrina Arshed, Mohammad Ansari, Puneet Sahgal, Abdalla Yousif

**Affiliations:** a Department of Internal Medicine, Raritan Bay Medical Center, 530 New Brunswick Ave., Perth Amboy, NJ, USA

**Keywords:** Myocarditis, Pericarditis, Myopericarditis, Ischemia, Myocardial, Diagnosis

## Abstract

Myocarditis can present in many different forms and can be overlooked by more life-threatening conditions. At times it may mimic conditions such as acute myocardial infarction and although it may have features highly suggestive of myocarditis, other etiologies need to be excluded. Thus, due to its clinical presentation, lab findings, and electrocardiogram analysis, it often can be confused with other conditions, making it a diagnostic dilemma of uncertainty. Myopericarditis is normally caused by viral infections, most common of which is coxsackievirus. Here we report a case of a 52-year-old gentleman who presented with a clinical picture of acute myocardial ischemia versus dissection, which overlooked a rather less threatening etiology of myopericarditis.

## Introduction

As an acute inflammatory disease that mainly impacts the myocardium, acute myopericarditis can be caused by viruses, systemic conditions, drugs, or toxins. Pericarditis and myocarditis at times can present together and coexist as they share common underlying causes such as coxsackievirus [1-3]. The dilemma arises however in the diagnosis of this disorder as it mimics myocardial infarction and in this case report also had features of acute aortic dissection and pulmonary embolism. Few reports have demonstrated myopericarditis presenting not only as acute myocardial infarction but also a symptomatic picture suggesting aortic dissection.

## Case Report

A 52-year-old male with a past medical history of hypertension, hypercholesterolemia, and smoking presented to the emergency room with complaints of chest pain, insidious in onset, and getting progressively worse. The pain was relieved when lying on his back and radiated to the left side of his chest, arm, and upper back. The patient stated the pain was a 10/10 and felt like a tearing pain down his back. Initially, patient believed the symptoms were secondary to acid reflux and took Tums. Overnight, the symptoms failed to resolve and he thus presented to the hospital. Patient denied any palpitations but had shortness of breath upon deep inhalation.

**Figure 1:**
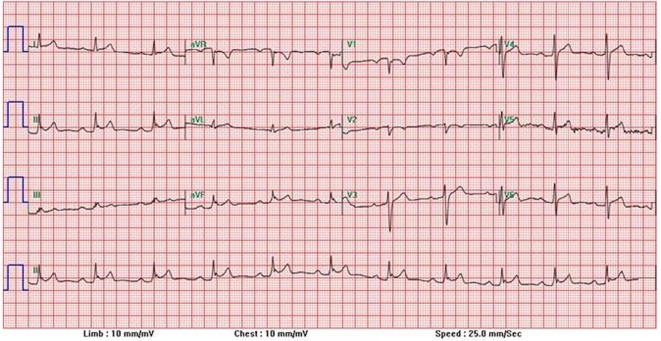
12-Lead EKG demonstrating diffuse ST elevations.

Vital signs demonstrated a blood pressure of 159/89, heart rate of 107, respiratory rate of 19, temperature of 98 °F, and pulse oximetry of 93% saturation. Physical examination findings were significant for only chest wall tenderness upon palpation and the patient was in clear distress and discomfort secondary to the severity of his chest and back pain. No friction rub was evident. Initial laboratory data showed a hemoglobin of 14.1, hematocrit of 43.1, white blood cell count of 10.4, and platelets of 230. Basic metabolic panel illustrated a sodium of 140, potassium of 4.4, chloride of 98, bicarbonate of 30, blood urea nitrogen of 24, creatinine of 1.7, and glucose of 99. His CKMB was 9.41 and his first troponin was elevated at 11.54. EKG (Fig. 1) illustrated ST elevations in multiple leads and thus Code Heart was called and patient was taken for cardiac catheterization.

**Figure 2:**
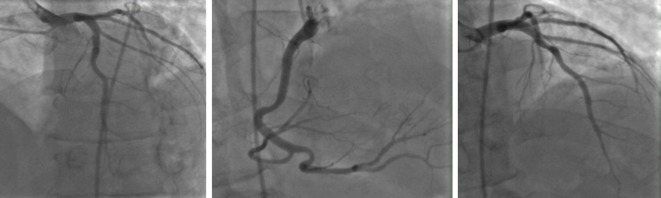
Cardiac catheterization illustrating patent arteries without any signs of occlusion or ischemia.

**Figure 3:**
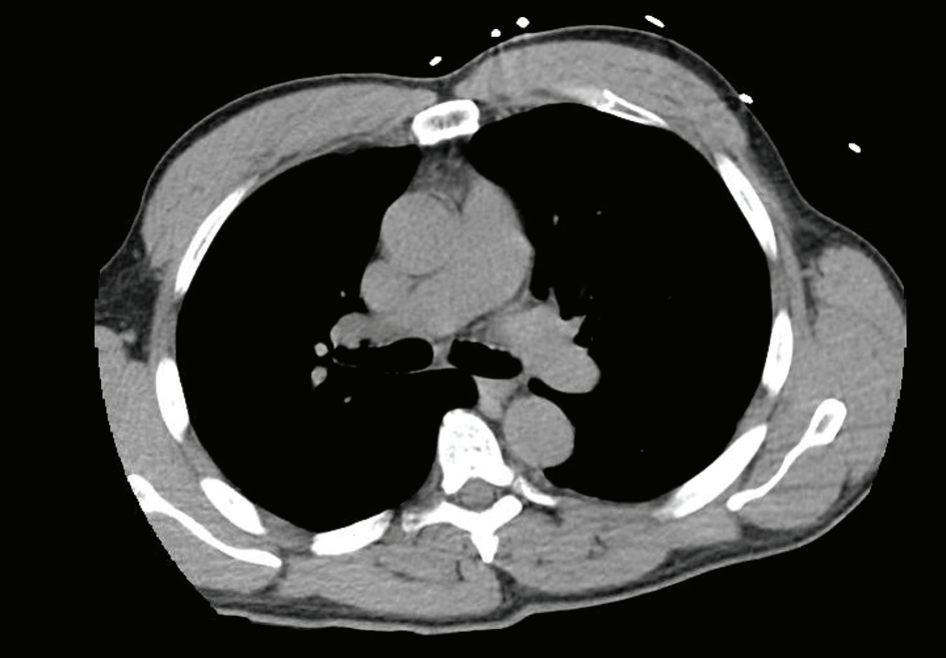
CT angiogram conducted showing absence of pulmonary embolism.

Cardiac catheterization revealed patent arteries and no occlusions were noted (Fig. 2). Post catheterization, patient continued to remain tachycardic and in significant distress from back pain. Aortic dissection and pulmonary embolism remained in the differentials at the time. Due to an elevated creatinine, a ventilation/perfusion (V/Q) scan was conducted while intravenous hydration was given. V/Q scan demonstrated indeterminate probability of pulmonary embolism. The following day with improvement in his creatinine, the patient underwent computed tomography angiography (CTA), which concluded that there was negative for dissection or embolism (Fig. 3). The clinical picture of the patient over the next few days improved with the use of NSAIDs. Serology later returned suggesting the presence of Coxsackie B virus.

## Discussion

Although there are multiple causes of myocarditis, pericarditis, and myopericarditis, viruses are one of the most common (Table 1). Coxsackie B virus, as seen in our patient, is the most commonly associated viral cause of myocarditis.

**Table 1:** List of Viral Causes of Myocarditis

Adenovirus
Coronavirus
Coxsackie virus (A, B)
Cytomegalovirus
Epstein-Barr virus
Hepatitis B
Herpes simplex
HIV
Influenza (A, B)
Mumps
Rabies
Rubella
Rubeola
Varicella-Zoster virus

The diagnosis and management of acute myopericaditis as early as possible is important, however may be limited by symptoms and signs suggesting a different cardiopulmonary process. It is also important to distinguish pericarditis from myocarditis as myocarditis has significant complications such as ventricular arrhythmia, cardiac dilation, and cardiac collapse which can develop rather abruptly [4, 5].

The gold standard of diagnosis, though not used commonly, is endomyocardial biopsy. It remains limited in its role due to a poor sensitivity and is not commonly used unless the patient is not responding well to medical treatment [1]. Endomyocardial biopsy shows a sensitivity of 43-64% and an overall complication rate of 6% with a 0.4% incidence of death due to perforation [6]. Another diagnostic modality that has been used is radiolabeled antimyosin antibody which can identify myocarditis but is non-specific as it detects myocardial necrosis from any cause and has a sensitivity of 67% and specificity of 63% [7, 8]. A third option involves the use of cardiac magnetic resonance imaging (CMR). This modality can find myocardial edema and myocyte damage non-invasively showing a characteristic pattern of contrast enhancement in patients with myocarditis [8]. However, in patients with infraction, it will show sub-endocardial enhancement, rather than enhancement originating from the epicardium and sparing the sub-endocardial layer as seen in myocarditis [8]. Unfortunately, the use of these diagnostic tests is not at all times beneficial, not available at many facilities, and only necessary when medical management does not show a clinical improvement in the patient’s symptoms.

A diagnosis of myocarditis is essential with the exclusion of other conditions, as myocarditis can be fatal and also lead to dilated cardiomyopathy and chronic heart failure as well. A better understanding of the pathophysiology and destructive nature of myocarditis through heart muscle destruction and development of dilated cardiomyopathy is important as it may improve the ease of its diagnosis and decrease the need for other diagnostic procedures. With a better understanding, interventions such as cardiac catheterization, CT angiograms, biopsy, and other diagnostic tests can be avoided.

Viruses mediate toxicity through focal necrosis of myocytes in the absence of inflammatory cell infiltrate within 3 days of infection and NK cells, protective antiviral antibodies, and infiltrating macrophages clear the virus from the myocardium [9]. The dilemma arises however as many symptoms and lab studies show overlapping features of ischemia and myocarditis. Myocarditis can demonstrate a rise in creatine kinase and electrocardiographic abnormalities as well. Along with ST elevations that may be diffuse, diffuse T wave abnormalities are a frequent feature on 12 lead ECK, with even 20% of patients developing a left bundle branch block [10]. As many of these features overlap with findings found in acute myocardial ischemia, it becomes difficult for a physician to make a clinical judgment in whether cardiac catheterization as a method of exclusion is necessary.

### Conclusion

To summarize, patients with myocarditis present with several symptoms, clinical and laboratory findings that may coincide with other diagnoses such as ischemia. In our case report, due to the severity of the patient’s presentation, significant back pain, tachycardia, respiratory distress, and other diagnoses such as aortic dissection and pulmonary embolism also need to be excluded. Thus, awareness of myocarditis presenting similar to these other conditions, can help physicians avoid unnecessary examinations and rapidly correct the underlying cause to prevent long-term risks. However, it is vital to keep other conditions in mind as the risk of long-term complications can be detrimental to a patient’s outcome.
